# Clinical Profile, Radiologic Features, and Clinical Course of Tumefactive Demyelinating Lesions in Two Tertiary Medical Centers in the Philippines: An 11-Year Retrospective Study

**DOI:** 10.7759/cureus.109831

**Published:** 2026-05-28

**Authors:** Bernard Kean M Capinpin, Ludwig F Damian

**Affiliations:** 1 Department of Neurology, St. Luke's Medical Center, Quezon City, PHL; 2 Institute for Neurosciences, St. Luke's Medical Center, Quezon City, PHL

**Keywords:** multiple sclerosis, neuroimaging, neuroimmunology, neuromyelitis spectrum disorder, tumefactive demyelinating lesion

## Abstract

Tumefactive demyelinating lesions (TDLs) are demyelinating lesions that can mimic tumors on imaging by presenting with mass effect, edema, and ring enhancement. They may be associated with a central demyelinating disease such as multiple sclerosis, neuromyelitis optica, or myelin oligodendrocyte glycoprotein antibody-associated disease. We aimed to determine the clinical, laboratory, and radiologic features, as well as the prognosis, of eight adult patients with TDLs at two tertiary hospitals in the Philippines. We conducted a chart review of patients diagnosed with TDLs from January 2014 to July 2024. Eight patients were diagnosed with TDLs. Most were treated as cases of multiple sclerosis. One tested positive for anti-aquaporin 4 antibody, and another was treated as a case of acute disseminated encephalomyelitis. All patients were given methylprednisolone pulse therapy. The majority of patients had a monophasic course, with symptoms resolving at follow-up. Among Filipino patients, TDLs are rarely diagnosed but mostly have a favorable prognosis.

## Introduction

Tumefactive demyelinating lesions (TDLs) are rare tumor-like lesions caused by demyelinating disease. They are defined as measuring >2cm and are hyperintense on T2-weighted MRI [1]. They may present with a mass effect, edema, and ring enhancement, mimicking a primary neoplastic process. Thus, they may pose a diagnostic challenge for clinicians, and the diagnosis may occasionally require confirmation by brain biopsy.

While multiple sclerosis and its variants have traditionally been implicated in this disorder, other autoimmune diseases, including neuromyelitis optica spectrum disorder (NMOSD), MOG antibody disease (MOGAD), Baló concentric sclerosis, acute disseminated encephalomyelitis (ADEM), and autoimmune encephalitis, have also been reported to present with tumefactive lesions. TDLs have also been reported as part of a systemic autoimmune disease, such as systemic lupus erythematosus [2] or as a paraneoplastic entity [3].

A study by Cacciaguerra et al. has shown that, among demyelinating diseases, MOGAD has the highest incidence of TDLs, followed by NMOSD [4]. A study conducted in Thailand found that among patients with inflammatory demyelinating disease, the incidence of TDLs is 2.4% [5].

The objective of this study was to determine the clinical, laboratory, and radiologic features, as well as the prognosis, of adult patients with TDLs at two tertiary academic centers in the Philippines.

This work was previously presented as a poster at the World Congress of Neurology 2025 in Seoul, South Korea. It was published as a conference abstract in the Journal of Neurological Sciences, which published an earlier draft of this paper. The entire manuscript has not yet been previously published.

## Materials and methods

This was a retrospective descriptive study conducted in two tertiary academic medical centers in the National Capital Region, Philippines. Adult patients (>18 years old) who were identified to have TDLs at any time during their disease course were included. TDL was defined as a lesion with a mass-like appearance, measuring >2 cm, and showing T2-weighted hyperintensity. TDLs were clinically or histopathologically diagnosed by a board-certified neurologist after investigation and follow-up. We searched our database from January 2014 to July 2024, and we reviewed the medical records of the included patients. The study was reviewed and approved by the Institutional Ethics Review Committee of St. Luke's Medical Center, and all methods were performed in accordance with the guidelines and regulations.

The following demographic and clinical data were collected through retrospective chart review: sex, age at presentation, disease duration, presenting symptomatology, neurologic deficits, and relevant comorbidities. Radiologic characteristics reviewed on MRI included lesion size, number, location, morphology, enhancement pattern, and the presence of perilesional edema and restricted diffusion on diffusion-weighted imaging. Advanced imaging findings, when available, including magnetic resonance spectroscopy (MRS) and magnetic resonance perfusion (MRP), were likewise reviewed. Histopathologic findings from biopsy or surgical specimens, when available, were also evaluated.

Patients who underwent MRS were examined using a standardized institutional protocol involving proton MRS acquisition with both short and intermediate echo times (TE 35 ms and 144 ms). The region of interest was positioned at the center of the predominant lesion to optimize metabolite characterization and minimize partial volume effects. Metabolite peaks and ratios, including choline, creatine, N-acetylaspartate, and the presence of lipid-lactate peaks, were reviewed when reported.

Cerebrospinal fluid (CSF) studies included opening and closing pressure, CSF glucose, CSF protein, CSF total cell count and differential count, CSF cytology, CSF IgG index, and oligoclonal bands. Antibody testing for CSF/serum anti-aquaporin 4 and anti-myelin oligodendrocyte using either a live-cell-based assay, an inactivated cell-based assay, or an enzyme-linked immunosorbent assay was performed upon request. Other ancillary tests, such as systemic autoantibodies, ophthalmologic examinations, and spinal MRI, were also obtained.

Acute treatments such as high-dose corticosteroids, plasma exchange, and intravenous immunoglobulin, as well as long-term treatments such as immunomodulators, were documented. The disease course, whether the disease was monophasic or relapsing, was determined.

Continuous variables were presented as the mean with standard deviation (SD). Categorical variables were presented as percentages. Descriptive statistics were processed using SPSS Statistics version 29 (IBM Corp. Released 2022. IBM SPSS Statistics for Windows, Version 29.0. Armonk, NY: IBM Corp.).

## Results

From 2014 to 2025, a total of eight individuals were diagnosed with TDLs in both institutions. The average age of onset was 43.25 (range: 22-74; SD = 18.73). Half were female. Half of the cases had weakness as the initial presentation. One case developed optic neuritis three years prior to another symptom attributable to a TDL. One case tested positive for anti-aquaporin-4 antibody, and another showed imaging features compatible with ADEM. The summary of the cases is in Table 1.

**Table 1:** Summary of cases CSF: cerebrospinal fluid, NA: not available, NMOSD: neuromyelitis optica spectrum disorder, ADEM: acute disseminated encephalomyelitis

Age and sex	Clinical features			CSF profile						Antibodies	Associated disease	Histopathology	Treatment
	Symptomatology	Clinical course	Comorbidities	Opening pressure (cmH2O)	Closing pressure (cmH2O)	CSF glucose (mg/dL)	CSF glucose ratio	Leukocytes	Protein (mg/dL)				
22/F	Nystagmus	Monophasic	None	(-)	(-)	(-)	(-)	(-)	(-)	(+) ANA, (-) anti-SSA, anti-SSB	(-)		Methylprednisolone pulse therapy
23/M	Hemiparesis	Monophasic	None	(-)	(-)	(-)	(-)	(-)	(-)	(-) ANA, anti-SSA, anti-SSB, p-ANCA, c-ANCA	(-)		Methylprednisolone pulse therapy
30/M	Hemiparesis	Monophasic	Hyperurecemia, impaired fasting glucose, dyslipidemia	28	20	64	0.60	4 (all lymphocytes)	29	(-) ANA	(-)		Methylprednisolone pulse therapy
34/F	Hemiparesis	Monophasic	None	(-)	(-)	(-)	(-)	(-)	(-)	(+) anti-aquaporin 4, (-) ANA, anti-SSA, anti-SSB	NMOSD		Methylprednisolone pulse therapy, rituximab
39/M	Hemiparesis	Monophasic	Optic Neuritis	(-)	(-)	(-)	(-)	(-)	(-)	(-) ANA	(-)	Parenchymal perivascular histiocytic infiltrates and perivascular chronic inflammation	Methylprednisolone pulse therapy, rituximab
59/F	Dysphagia	Monophasic	Hypertension	(-)	(-)	(-)	(-)	(-)	(-)	NA	(-)		Methylprednisolone pulse therapy
65/F	Headache	Monophasic	Hypertension, dyslipidemia	7	6	57	0.59	12 (all lymphocytes)	55	NA	(-)		Methylprednisolone pulse therapy
74/F	Dizziness	Relapsing	Hypertension, diabetes	12	8	105	0.72	8 (all lymphocytes)	85	(-) ANA, anti-SSA, anti-SSB	ADEM		Methylprednisolone pulse therapy; IV immunoglobulin

**Figure 1:**
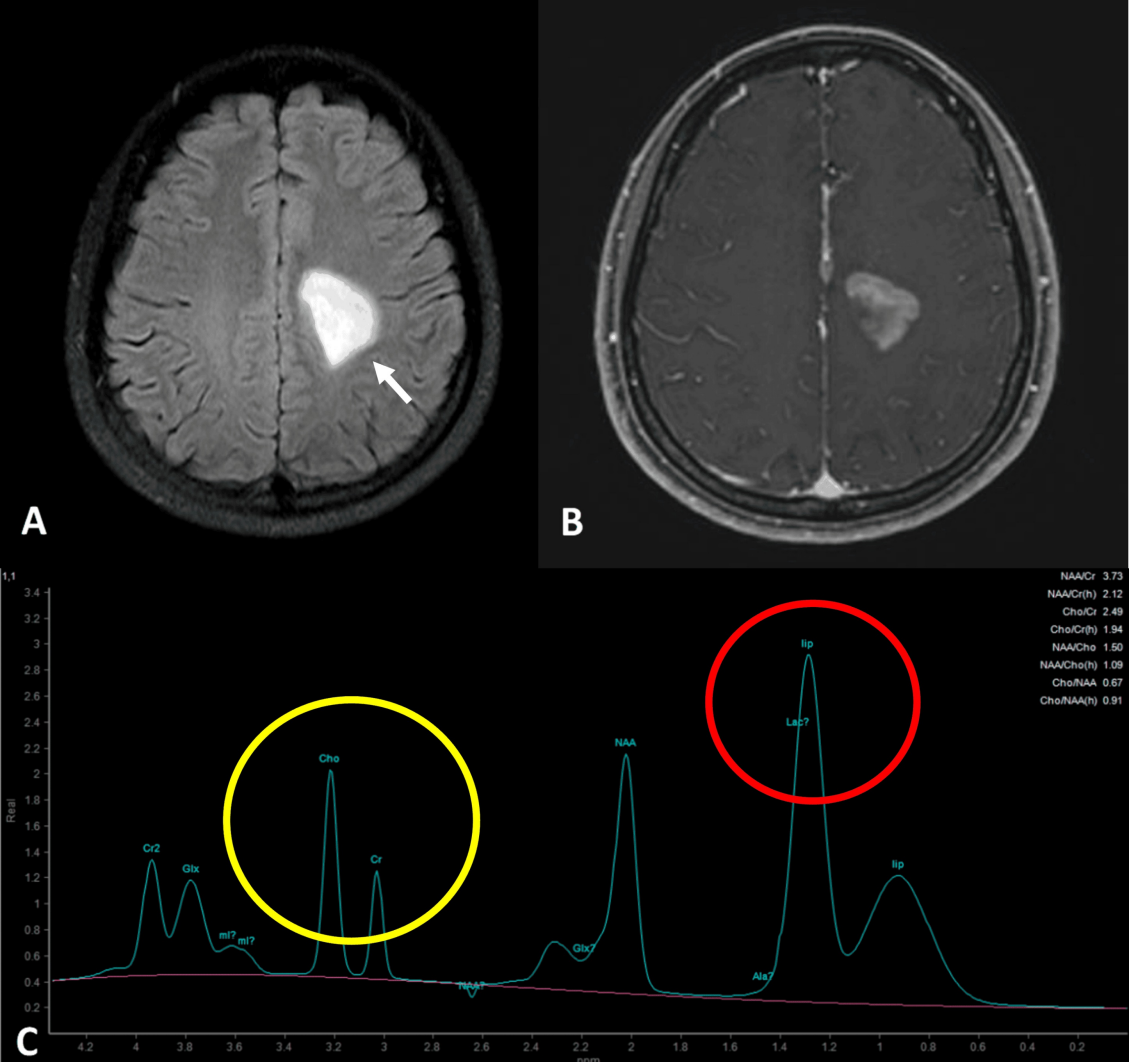
34/F who presented with right-sided weakness and tested positive for anti-aquaporin 4 antibody. (A) Plain FLAIR sequence showed perilesional edema (white arrow). (B) T1-weighted contrast study showed open-ring enhancement. (C) MRS revealed elevated choline to creatinine ratio (yellow circle) and elevated lipid-lactate peak (red circle) F: female, FLAIR: fluid-attenuated inversion recovery, MRS: magnetic resonance spectroscopy

All patients underwent 3-Tesla MRI, MRS, and MRP at our institution. On MRI, the average lesion size was 3.71 cm (SD = 2.29). Most had multifocal lesions. The majority of the lesions were supratentorial, with the frontal and parietal lobes being the most affected. Lesions could also be seen infratentorial, such as in the cerebellum, pons, and medulla. One patient had radiographic evidence of a unilateral optic neuritis, showing enhancement along the intracanalicular and intracranial segments of the optic nerve. One patient had juxtacortical white matter lesions. Five of the TDLs demonstrated the classic partial ring enhancement, while two had heterogenous enhancement. Three cases (37.5%) showed perilesional edema, while three cases (50%) had restricted diffusion on DWI/ADC-weighted sequences. Seven out of eight patients had undergone the standardized MRS at our institution. However, numerical values for the spectroscopy study of one patient could not be retrieved. All cases with MRS showed an elevated choline-to-creatinine ratio. Six had elevated lactate peaks (85.71%). See Table 2 for a summary of findings and Figure 1 as a representative example.

**Table 2:** Summary of MRI findings Normal parameters: choline/creatinine ratio: <1. NAA/choline ratio: 1.6-2.5. Absent lipid-lactate peak. Glutamate-glutamine/creatinine ratio: <0.5. MRI: magnetic resonance imaging, MRS: magnetic resonance spectroscopy, MRP: magnetic resonance perfusion, TDL: tumefactive demyelinating lesion, rCBV: relative cerebral blood volume, ASL: arterial spin labeling, NAAN: acetylaspartate, NA: not available, F: female, M: male, ↑: elevated, ↓: decreased

Age and sex	Radiologic features							Features mimicking neoplasm/infection	Imaging features favoring TDL/demyelination
	Largest diameter (cm)	Location	Edema	Restricted diffusion	Enhancement	MRS and MRP	Other MRI findings		
22/F	2.9	Pontomesencephalic junction	(-)	(-)	Faint enhancement	Choline/creatinine ratio: 2.5 (↑); (+) mildly elevated lactate peak; hypoperfusion on arterial spin labelling	(-)	Elevated choline/creatinine peak	Lack of edema and mass effect; hypoperfusion on arterial spin labeling; decreased enhancement on follow-up MRI following steroid treatment
23/M	8.2	Frontoparietotemporal lobes, basal ganglia	(+)	(+)	Incomplete ring enhancement	Choline/creatinine ratio: 1.6 (↑); (+) elevated lactate peak; peripheral hyperperfusion with hypoperfused core	(-)	Prominent edema and mass effect	Incomplete ring enhancement
30/M	2.1	Centrum semiovale	(-)	(+)	Incomplete ring enhancement	NA	(-)	Mass effect	Incomplete ring enhancement; interval decrease in size and enhancement after steroid therapy
34/F	2.7	Frontoparietal, parieto-occipital	(+)	(+)	Incomplete ring enhancement	Choline/creatinine ratio: 1.94 (↑); (+) moderately elevated lactate peak; increased rCBV	(-)	Increased rCBV	Incomplete ring enhancement; interval decrease in size and enhancement after steroid treatment
39/M	3.6	Temporal, parietal	(-)	(-)	Heterogenous enhancement	Choline/creatinine ratio: 1.28 (↑); N-acetylaspartate NAA/choline ratio: 1.27 (↓); (+) mildly elevated lipid-lactate peak	Right temporal periventricular region and left frontal juxtacortical white matter hyperintensities	Mass effect	Juxtacortical lesions
59/F	4.5	Cerebellar peduncle	(-)	(+)	Incomplete ring enhancement	Choline/creatinine ratio: 1.19 (↑); N-acetylaspartate NAA/choline ratio: 0.98 (↓); (+) markedly elevated lipid-lactate peak; glutamate-glutamine/creatinine ratio: 0.8 (↑)	Enhancement of the intracanalicular to the intracranial segments of the right optic nerve	Increased rCBV	Incomplete ring enhancement; findings suggestive of optic neuritis
65/F	2	Thalamus	(-)	(-)	Heterogenous enhancement	Choline/creatinine ratio: 1.25 (↑); (+) lipid-lactate peak; glutamate-glutamine/creatinine ratio: 0.7; (↑) Increased rCBV	(-)	Heterogenous enhancement; increased perfusion on arterial spin labeling	Regression of size and decreased perfusion on ASL on MRI following steroid treatment
74/F	3.7	Frontoparietal, cerebellum, medulla	(+)	(+)	Incomplete ring enhancement	Increased choline/creatinine ratio; Increased NAA peak	(-)	Mass effect	Incomplete ring enhancement: clinical and radiologic stability after steroid treatment

**Figure 2:**
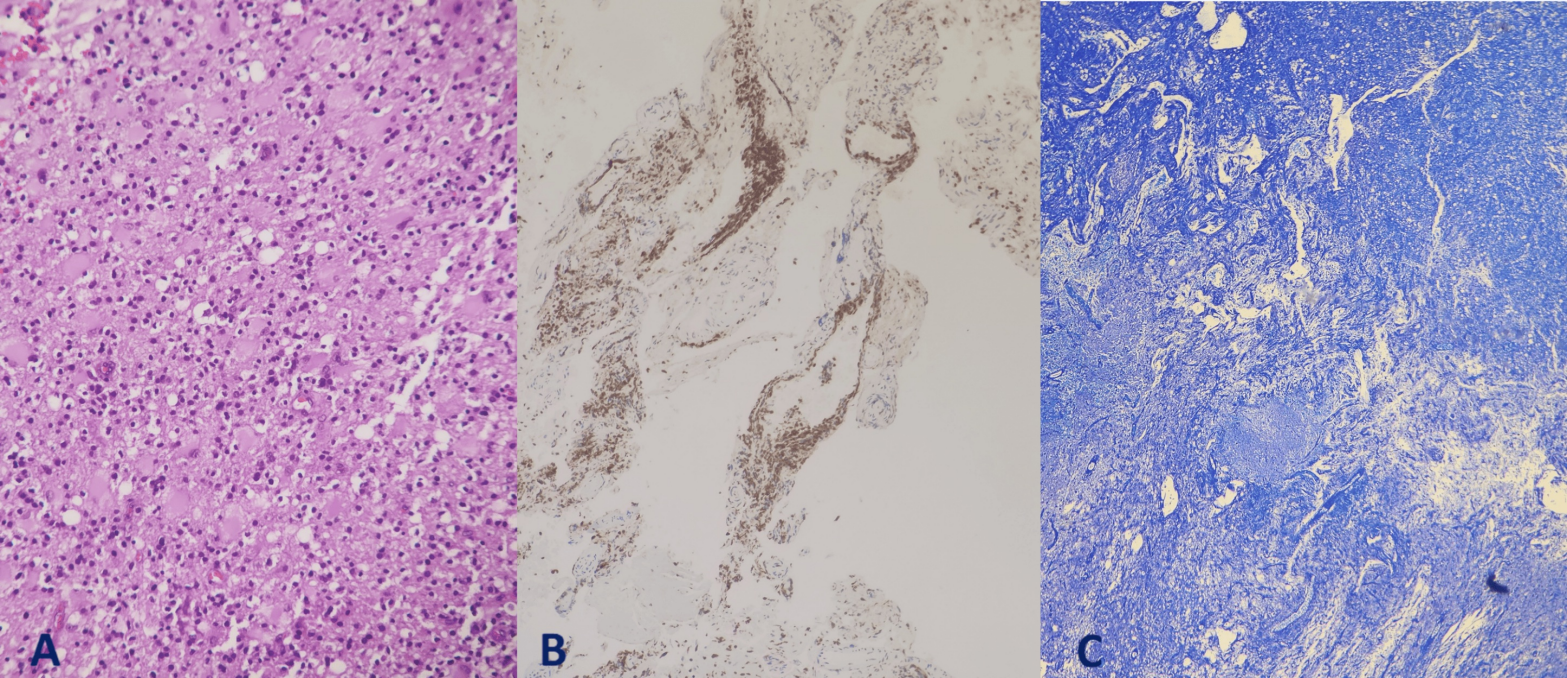
23/M who presented with left-sided weakness. Biopsy of a frontoparietal mass was done. (A) Hematoxylin and eosin showed lymphocyte-rich tissue. (B) CD3 immunohistochemistry stain showing perivascular T-lymphocytes. (C) Luxol fast blue staining showed no intense blue staining, indicating the absence of myelin M: male

Only three patients underwent lumbar puncture, which may be due to concerns for herniation due to the mass effect of the lesion. One out of the three had an elevated opening pressure at 28 cmH₂O. Two had elevated CSF protein levels and pleocytosis. Oligoclonal bands were negative. One tested positive for antinuclear antibody. Two patients underwent biopsy of the tumefactive lesion. One of the patients had a heterogenous pattern of enhancement, prompting suspicion of a neoplastic process. The other patient had undergone a biopsy to rule out an aggressive tumor since the lesion demonstrated prominent edema as well as mass effect in the background of peripheral hyperperfusion. The absence of intense blue staining on Luxol fast blue confirmed the diagnosis histologically. Both tissue specimens had histiocyte-rich parenchyma and prominent perivascular lymphocytic infiltrates (Figure 2).

Although no standardized protocol existed across institutions for the treatment of TDLs, all patients received intravenous methylprednisolone 1000 mg for five days during the acute phase. One patient underwent plasmapheresis due to poor response to the initial pulse steroid therapy. Two patients subsequently underwent rituximab infusion. One of these patients tested positive for anti-aquaporin 4 and was diagnosed with NMOSD. Another underwent rituximab infusion due to a history of optic neuritis and presence of juxtacortical white matter lesions, which raised a suspicion for a CNS demyelinating disease. However, he did not fit any of the criteria. All patients had at least one year of follow-up, though clinical or radiologic post-treatment response was not predefined. Most patients had a monophasic course of illness, and deficits had resolved on follow-up (n = 7, 87.5%), while one had relapsed after five months.

## Discussion

As TDL is a rare and frequently misdiagnosed disease, there is a need to understand its clinical features and course. Our study showed no sex preference for the disease, unlike other large cohorts that reported a slight female preponderance (1.3:1) [6-7]. The mean age at presentation is older than in other cohorts, typically in the 30s [7-8]. The onset of TDLs is not limited to younger age groups; older adults have been reported to develop TDLs. They are more likely to undergo biopsy and have poorer Expanded Disability Status Scale scores on follow-up [9]. The predominance of motor deficits in our cohort likely reflects the preferential involvement of the frontoparietal white matter, a pattern also observed in other large cohorts [8]. This shows that lesion location strongly influences initial clinical presentation.

One of the most clinically challenging aspects of TDLs is their close radiologic resemblance to neoplastic and infectious intracranial lesions. TDLs commonly mimic glioma, lymphoma, metastasis, or abscess because they may present as large space-occupying lesions with edema, mass effect, and contrast enhancement. Several imaging characteristics may favor demyelination over neoplasia. While there are several features suggestive of TDLs, on standard contrast MRI, TDLs can be varied radiologically, showing different patterns of enhancement. Open-ring or incomplete rim enhancement, particularly with the incomplete portion directed toward the cortex, is considered highly suggestive of TDL. In several large cohorts, the open-ring enhancement is the most common finding [6,8,10-11]. This enhancement pattern may increase the likelihood and diagnostic certainty that a tumefactive lesion is demyelinating [12]. A meta-analysis of MRI findings in TDLs showed that this enhancement pattern is highly specific, with 98-100% specificity [13]. The enhancing area represents active demyelination in the white matter, while the incomplete portion represents either grey matter or chronic inflammation [14]. Other supportive features include relatively mild edema and mass effect, T2 hypointense rims, peripheral rather than central diffusion restriction, low cerebral blood volume on perfusion imaging, and interval reduction following corticosteroid therapy.

For lesions not presenting with this typical pattern, diagnosis may be more challenging, and further diagnostics may be necessary. Three of the patients presented with either heterogenous or faint enhancement. One had other findings of juxtacortical lesions, which suggested a demyelinating disease, and the patient underwent biopsy, which confirmed the diagnosis. Another had an elevated glutamate-glutamine ratio, which favored a diagnosis of TDLs. Lumbar puncture was done, showing lymphocytosis, but CSF studies were negative for malignancy or infection. In another case, arterial spin labeling was done, showing hypoperfusion, which favored a non-neoplastic lesion. As the patient did not present with systemic signs of infection, empiric steroid treatment was administered, and a subsequent MRI showed regression of enhancement and size. In these cases, adjunctive diagnostics may be necessary to support the diagnosis of a TDL.

MRS was pursued in most of our cases, and all showed an elevated choline/creatinine ratio. Previous studies have shown that this spectroscopic finding is common in patients with TDLs; elevated choline or lactate peaks are not specific for TDLs and may also be present in neoplasms [15]. Some studies have shown that gliomas have a higher choline/NAA ratio than TDLs, and a ratio greater than 1.72 may differentiate a glioma from a TDL [16]. On MRP, TDLs typically have lower rCBV values than high-grade gliomas, even gliomas or lymphoma [17]. However, hyperperfusion may also be present in TDLs on MRS, indicating highly active inflammatory areas [18].

Other intracranial findings may also support a demyelinating etiology for tumefactive lesions. One patient with a biopsy-confirmed TDL presented with juxtacortical lesions on the initial MRI and follow-up imaging. While non-specific, juxtacortical white matter lesions may raise suspicion for demyelinating disease such as multiple sclerosis, in addition to cranial imaging findings, some patients may have underlying myelitis [19] and optic neuritis [20]. The coexistence of optic neuritis and myelitis in some patients supports the possibility that TDLs may occur within a broader spectrum of inflammatory demyelinating disorders. This underscores the importance of interpreting the entire imaging dataset to develop an accurate diagnosis.

While not all diagnosed cases of TDLs are confirmed by biopsy, and diagnosis is made by integrating clinical presentation, radiologic features, response to empiric treatment, and behavior over time, biopsy plays an important role in atypical cases [21]. Our two histopathologically confirmed cases are consistent with published reports. In a systematic review of biopsy-confirmed cases, the main histopathologic findings were perivascular and parenchymal infiltrates predominantly comprised of T-cells and foamy macrophages [22]. Demarcation of the demyelinated tissue from the preserved white matter can be highlighted by Luxol fast blue staining [23]. In a review by Vakrakou et al., idiopathic or multiple sclerosis-associated TDLs are associated with a confluent pattern of demyelination and high phagocytic activity at the edge of the lesion [24]. This correlates with the open-ring enhancement pattern, where the active areas of inflammation and demyelination are at the edges of the lesion. T-lymphocytes, rather than B-lymphocytes, were the predominant infiltrates in a study of atypical demyelinating lesions [25]. Reactive astrocytes called Creutzfeldt-Peters cells may also be seen in TDLs, although this is not a specific finding and can also be seen in glioblastoma. None were seen in our cases.

Due to its rarity, optimal treatment strategies have yet to be established, although small case reports suggest that it is responsive to immunomodulatory treatment and has a benign course [7]. Corticosteroids remain the first-line therapy in this cohort, as well as others. Few patients had proceeded with disease-modifying treatments such as rituximab to prevent relapse. This may be reasonable when underlying relapsing demyelinating disease associated with TDLs is suspected. Rituximab has been reported to be used for steroid-refractory cases [26] and for maintenance therapy [5]. Nonetheless, for some of our patients, there was no recurrence of deficits regardless of whether immunomodulators were initiated. Further studies on disease-modifying therapy for TDLs are warranted, especially given their potential to lie on a spectrum with other demyelinating diseases.

Studies have also demonstrated the heterogenous behavior of TDLs. Some studies have shown that it is mainly a monophasic illness with rare recurrence, while other studies have shown a high rate of relapse [27]. The predominantly monophasic course observed in our cohort may suggest a higher proportion of idiopathic or non-multiple-sclerosis-associated TDLs. Most studies on TDLs have been conducted in locations where multiple sclerosis is a common central nervous system demyelinating disorder. In the Philippines, the incidence of multiple sclerosis is unknown [28], as well as TDLs. The varying incidence of central nervous system demyelinating disorders across geographic regions may contribute to the relapse rate of TDLs.

The study is limited by its retrospective design and the relatively small sample size, reflecting the rarity of TDLs. CSF studies and serologic testing for antibodies associated with demyelinating diseases, including anti-aquaporin-4 and anti-myelin oligodendrocyte glycoprotein antibodies, were not routinely performed in all patients. In many cases, lumbar puncture was initially deferred because of concern for possible cerebral herniation, as these patients commonly presented with large space-occupying lesions on neuroimaging. However, our experience suggests that lumbar puncture can be safely performed in carefully selected patients with TDLs, and that CSF analysis remains an important diagnostic modality for excluding infectious, neoplastic, and other inflammatory mimickers.

Access to antibody testing may be limited by cost and availability, particularly in resource-constrained settings, leading to misclassification of cases. This is particularly relevant given that TDLs may occur in a spectrum of demyelinating disorders, including multiple sclerosis, NMOSD, and MOGAD. Previous studies also suggest geographic and ethnic variability in the underlying etiologies of TDLs. In a predominantly Caucasian cohort, TDLs have been more frequently associated with multiple sclerosis [8], whereas studies from Asian populations have reported a higher proportion of NMOSD-related cases [5]. Because treatment strategies and prognosis differ across these disease entities, testing for AQP4-IgG and MOG-IgG antibodies is recommended whenever feasible to establish the underlying diagnosis and guide disease-specific management.

## Conclusions

This study represents the largest reported cohort of TDLs in the Philippines, contributing valuable data from a region where such reports remain limited. TDLs are rare but clinically significant entities that pose substantial diagnostic challenges, frequently mimicking neoplastic and infectious processes due to overlapping radiologic features. Among imaging characteristics, the presence of an open-ring pattern of contrast enhancement remains one of the most suggestive findings, having been consistently reported as more common than closed-ring enhancement in prior studies.

The clinical course in our cohort was predominantly monophasic, with a low rate of relapse, although variability in long-term outcomes has been described in the literature. High-dose intravenous methylprednisolone remains an effective first-line therapy, with most patients demonstrating a favorable clinical and radiologic response, consistent with existing evidence. The retrospective design and relatively small sample size limit the generalizability of our findings. Future prospective multicenter studies with longer follow-up periods are needed to better define the natural history, prognostic factors, and optimal management strategies for TDLs in the local population.
